# Prospective evaluation of interrater agreement between EEG technologists and neurophysiologists

**DOI:** 10.1038/s41598-021-92827-3

**Published:** 2021-06-28

**Authors:** Isabelle Beuchat, Senubia Alloussi, Philipp S. Reif, Nora Sterlepper, Felix Rosenow, Adam Strzelczyk

**Affiliations:** 1 Epilepsy Center Frankfurt Rhine-Main and Department of Neurology, Center of Neurology and Neurosurgery, Goethe-University Frankfurt, Schleusenweg 2-16 (Haus 95), 60528 Frankfurt am Main, Germany; 2 LOEWE Center for Personalized Translational Epilepsy Research (CePTER), Goethe-University Frankfurt, Schleusenweg 2-16, 60528 Frankfurt am Main, Germany; 3 Department of Neurology, Ortenau Klinikum, Ebertplatz 12, 77654 Offenburg, Germany

**Keywords:** Encephalopathy, Epilepsy

## Abstract

We aim to prospectively investigate, in a large and heterogeneous population, the electroencephalogram (EEG)-reading performances of EEG technologists. A total of 8 EEG technologists and 5 certified neurophysiologists independently analyzed 20-min EEG recordings. Interrater agreement (IRA) for predefined EEG pattern identification between EEG technologists and neurophysiologits was assessed using percentage of agreement (PA) and Gwet-AC1. Among 1528 EEG recordings, the PA [95% confidence interval] and interrater agreement (IRA, AC1) values were as follows: status epilepticus (SE) and seizures, 97% [96–98%], AC1 kappa = 0.97; interictal epileptiform discharges, 78% [76–80%], AC1 = 0.63; and conclusion dichotomized as “normal” versus “pathological”, 83.6% [82–86%], AC1 = 0.71. EEG technologists identified SE and seizures with 99% [98–99%] negative predictive value, whereas the positive predictive values (PPVs) were 48% [34–62%] and 35% [20–53%], respectively. The PPV for normal EEGs was 72% [68–76%]. SE and seizure detection were impaired in poorly cooperating patients (SE and seizures; p < 0.001), intubated and older patients (SE; p < 0.001), and confirmed epilepsy patients (seizures; p = 0.004). EEG technologists identified ictal features with few false negatives but high false positives, and identified normal EEGs with good PPV. The absence of ictal features reported by EEG technologists can be reassuring; however, EEG traces should be reviewed by neurophysiologists before taking action.

## Introduction

Electroencephalograms (EEGs) are widely performed for a large range of indications, including but not restricted to epilepsy and seizure diagnosis and classification^1,2^, status epilepticus (SE) diagnosis and treatment monitoring^3–5^, the investigation of consciousness disorders^6^, and prognostication after cardiac arrest^7^. Although a precise glossary exists to describe the most common EEG features^8^, and the American Society of Clinical Neurophysiology (ACNS) has established standardized criteria for intensive care unit (ICU) EEG analysis^9^, EEG interpretation remains subjective and is likely affected by the interpreter’s level of training, location of training, and the patient’s history. Several studies have examined the interrater agreement (IRA) of EEG interpretations after the introduction of the ACNS criteria, especially in ICU settings between experts^10–13^. However, evidence regarding the IRA between certified EEG readers and EEG technologists is currently lacking.

The principal role of EEG technologists is to obtain high-quality recordings. However, their contributions often extend beyond this role. For example, EEG technologists were reported to make a significant contribution to the diagnosis of childhood epileptic syndrome by collecting clinical information during the preparation of children for EEGs^14^. Technologists’ roles are not limited to EEG recording; they may also perform EEG readings. EEG technologists are often the first to read the EEGs at the patient’s bedside and are expected to contact the physicians in charge in case of findings requiring urgent action. Furthermore, with the increasing use of continuous EEG (cEEG) monitoring, EEG technologists are often asked to review EEG monitoring^15^. A national survey assessing cEEG ICU indications and procedures at 151 institutions in the United States revealed that EEG technologists reviewed 100% of the cEEG records at 26% of institutions and half of the records at 56% of institutions^15^.

In a large, prospective, single-center cohort, we aimed to investigate the IRA between certified neurophysiologists and EEG technologists. We also aimed to investigate which clinical factors, if any, may alter the EEG-reading performances of EEG technologists.

## Methods

### Study design

This single-institution, prospective cohort study, was approved by Ethics Commission of Goethe-University Frankfurt (number 278/15) and registered at the German Clinical Trials Register (DRKS Trial Number: DRKS00009863; Universal Trial Number: U1111-1178-2516; Registered 13/01/2016, http://www.drks.de/ DRKS00009863). Informed consent was waived due to the anonymized analysis of EEG and clinical data. The study was performed in accordance with relevant guidelines and regulations.

### Patients

Consecutive adults and adolescents (≥ 15 years old) who underwent routine 20-min EEG (rEEG) recordings during a one-year period, starting January 2016, were included. With the exception of EEGs recorded for brain death diagnoses^16^, any routine EEGs that were recorded at our neurology department during the study period with a minimum duration of 20 min were included, regardless of the clinical indication for the recording or the patient´s localization (ambulatory, neurological ward, ICU or other medical and surgical wards). The indications for recordings were dichotomized between: (1) epileptic indications included SE, seizure or seizure suspicion, and the management of known epilepsy, such as the worsening of seizures or driving wishes; and (2) nonepileptic indications included disorders associated with consciousness or delirium. The final diagnosis was prospectively defined according to the International League Against Epilepsy (ILAE) criteria^2^: (1) no epilepsy-related diagnosis; (2) known epilepsy; (3) new epilepsy diagnosis; (4) first seizures; and (5) acute symptomatic seizures. Seizures and epilepsy types were prospectively classified according to the ILAE definitions^1, 2^. Status epilepticus (SE) was defined according to the current guidelines as seizures that persist for longer than 5 min or EEG patterns consistent with non-convulsive SE, as previously defined^3,4,17^. Collaboration was assessed by EEG-technologists during the EEG recordings and subjectively categorized into good/moderate/poor.

### EEG recordings and analysis

EEGs were recorded using at least 21 electrodes, arranged according to the international 10–20 system. Reduced montages were allowed in neurosurgical patients, consistent with common practices, and more extensive montages were possible.

A standardized EEG interpretation sheet was provided to EEG technologists and neurophysiologists. The analyzed parameters included: (1) the presence of sleep figures (K complexes and spindles); (2) posterior dominant rhythm (PDR) presence and main frequency; (3) the presence of a slowing with the characteristics of (3a) slowing localization: focal, hemispheric, or generalized and (3b) slowing duration: intermittent or continuous; (4) the presence of interictal epileptiform discharges (IED) with the characteristics of (4a) IED localization: focal, multifocal, or generalized; (5) the occurrence of seizures; (6) SE; and (7) Global conclusion, dichotomized as normal or pathologic. The EEG interpretation sheet (Supplementary Fig. 1) was first filled by the EEG technologists at the bedside, during EEG recordings, and then by the neurophysiologists, who were blinded to the technologists’ conclusions, during formal EEG readings. A total of 8 EEG technologists, who each had at least three years of training in neurophysiological diagnostics, as “Medizinisch-Technische/r Assistent/in–Funktionsdiagnostik” (MTA-F), and 5 trained neurophysiologists, with board certifications in EEG readings (including PSR, FR, and AS), participated in this study.

EEG readings were performed under “real-life” conditions, with no additional training provided to the EEG technologists before the study. The patients’ clinical data, such as the reason for referral and medical history, including previous seizures or known epilepsy diagnoses, were available to the EEG readers.

### Statistical analysis

Continuous and categorical variables are reported as the medians and range and were compared using Wilcoxon or T-tests, as appropriate. Binary variables are reported as the number and percentage and were assessed by Chi-square or Fisher’s exact tests and post hoc, pairwise Fisher’s exact tests. The predictive performances of EEG technologists for the identification of each predefined EEG pattern, when considering the neurophysiologists’ conclusions to be the gold standard, were estimated using exact binomial distributions, and the results are presented as specificity (Sp), sensitivity (Se), positive predictive value (PPV), or negative predictive value (NPV), with 95% confidence intervals (95% CIs). Exams during which EEG technologists reported the specified EEG features as being present when neurophysiologist reported the features as being absent were considered false positives (FPs), whereas those in which neurophysiologists coded the EEG features as being present but the EEG technologists coded the features as being absent were considered false negatives (FNs). The FP percentages are expressed as the FP rate among all exams coded “yes” by the EEG technologists, whereas the FN percentages are expressed as the FN rate among all exams coded “no” by the EEG technologists. Interrater agreement (IRA) was calculated using the Gwet’s agreement coefficient AC1 (for categorical dta) and AC2 (for ordinal data) to avoid the kappa paradox^18^ and the percentage of agreement (PA). The recommended strength of agreement nomenclature was used: < 0 = Poor; 0–0.20 = Slight; 0.21–0.40 = Fair; 0.41–0.60 = Moderate; 0.61–0.80 = Substantial; 0.81–1.0 = Almost perfect^19^. The Benjamini–Hochberg (BH) procedure was applied to control for the false-discovery rate, using a q-value of 0.05.

Data analyses were performed using IBM SPSS statistics software, version 27.0 (IBM Corporation, Armonk, New York; USA), and R, version 4.0.0 (R Foundation for Statistical Computing, Vienna, Austria). The figure is presented in a color-blind friendly color-scheme^20^.

### Ethics approval

This prospective study was approved by the ethical committee of the medical faculty of the Goethe-University Frankfurt.

## Results

The trial included 1528 EEG recordings. The patients’ median age was 55 years (IQR 21.5–88.5; range 15–95), and 51.1% were women (Table 1). The majority of the EEG recordings (1039, 68%) were obtained from very collaborative patients and contained minimal artifacts. EEGs were performed primarily due to epilepsy in 698 cases (45.7%). A total of 909 (59.5%) recordings were performed in patients who had experienced at least one epileptic seizure, and 619 (40.5%) patients did not have any epilepsy-related diagnoses. Among patients who had experienced at least one epileptic seizure, 497 had focal seizures: with temporal onset in 184, frontal onset in 139, parietal onset in 52, occipital onset in 12, multifocal in 57, and lateral onset without further lobe specification in 53; in the remaining 315 patients, seizure onset was unknown or not documented.

**Table 1:** Demographic and clinical characteristics of the total EEG cohort (n = 1528).

Age, median (range)	55 (15–95)
Female, n (%)	781 (51.1)
EEG recorded for “Epilepsy related” reasons, n (%)	698 (45.7)
History of craniotomy, n (%)^a^	328 (21.5)
Intubated during recording, n (%)	113 (7.4)
**Consciousness during EEGs recording, n (%)**^b^	
Fully awake	1116 (76.3)
Stuporous	107 (7)
Coma	120 (7.9)
**Collaboration during EEGs recoding, n (%)**	
Good	1039 (68.0)
Moderate	202 (13.2)
Poor	287 (18.8)
**Diagnosis, n (%)**^c^	
New Epilepsy diagnosis	147 (9.6)
First seizures	90 (5.9)
Acute symptomatic seizures	94 (6.2)
Known Epilepsy	578 (37.8)
Status epilepticus	183 (12)
**Epilepsy type, n/total patients with epilepsy diagnosis**	
Generalized	82/725
Focal	446/725
Unknown	197/725
**Seizure onset, n/total patients with seizures**	
Generalized seizure onset	97/909
Focal seizure onset	497/909
Unknown seizure onset	315/909

^a^One patient with missing data.

^b^Two patients with missing data.

^c^One EEG recording can be related to several categories.

**Figure 1:**
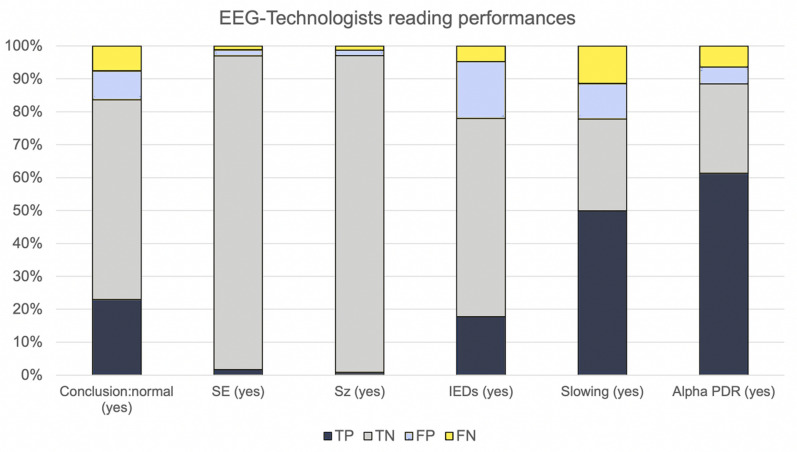
Percentages of true positives (in dark green; EEG patterns coded “yes” by both EEG technologists and neurophysiologists), true negatives (in light green; EEG patterns coded “no” by both EEG technologists and neurophysiologists), false positives (in orange; exams coded “yes” by EEG technologists and “no” by neurophysiologists), and false negatives (in red; exams coded “no” by EEG technologists and “yes” by neurophysiologists). *FN* False negative, *FP* false positive, *TP* true positive, *TN* true negative, *IEDS* interictal epileptiform discharges, *PDR* posterior dominant rhythm, *SE* status epilepticus, *Sz* seizures.

The measured IRA values between neurophysiologists and EEG technologists are shown in Table 2, and the proportions of true positive (TP), true negative (TN), false negative (FN), and false positive (FP) readings by the EEG technologies for the primary EEG feature is presented in Fig. 1. The IRA was scored “almost perfect” for seizure and SE detection (AC1 = 0.97 for both). The EEG technologists were able to diagnose these features with high negative-predictive value (NPV, for both, 99%; 95% CI 98–99%) but low positive predictive value (PPV, 35%; 95% CI 25–38% for seizures; 48%; 95% CI 43–74% for SE). Global conclusion, dichotomized as normal versus pathologic, demonstrated “substantial” IRA (AC1 = 0.71), and EEG technologists were able to identify normal EEGs with 72% (95% CI 68–76%) PPV and 89% (95% CI 87–91%) NPV. Globally, EEG technologists detected ictal elements with a very low FN rate and a high FP percentage (Table 2).

**Table 2:** Interobserver agreement and EEG technologists’ reading performances regarding the primary EEG features.

	Reported findings, N (%)		Interrater agreement (Gwet AC1)			EEGs technologists’ readings performances					
	EEG technologists	Neurophysiologists	PA (95%CI)	AC1 (95%CI)	Strength of IRA	FP, N (%)	FN, N (%)	Se, % (95% CI)	Sp, % (95% CI)	PPV, % (95% CI)	NPV, % (95% CI)
IEDs (yes)	537 (35.3)	344 (22.5)	78.0 (76–80)	0.63 (0.59–0.67)	Substantial	265 (49.3)	72 (7.3)	79 (74–83)	78 (75–83)	51 (46–55)	93 (93–94)
SE (yes)	54 (3.5)	44 (2.9)	97.0 (96–98)	0.97 (0.96–0.98)	Almost perfect	28 (51.9)	18 (1.2)	59 (43–74)	98 (97–99)	48 (34–62)	99 (98–99)
Seizures (yes)	37 (2.4)	33 (2.2)	97.1 (96–98)	0.97 (0.96–0.98)	Almost perfect	24 (64.7)	20 (1.3)	39 (23–58)	98 (98–99)	35 (20–53)	99 (98–99)
Slowing (yes)	930^a^ (60.9)	932^b^ (61.4)	77.1 (76–80)	0.58 (0.53–0.62)	Moderate	164 (17.6)	173 (29.0)	81 (79–84)	72 (68–76)	82 (80–85)	71 (67–75)
Sleep figures (yes)	245^c^ (16.3)	135^d^ (8.9)	84.1 (82–86)	0.79 (0.77–0.82)	Substantial	175 (71.4)	63 (5.0)	52 (43–61)	87 (85–89)	28 (23–34)	95 (94–96)
Alpha PDR (yes)	1014^a^ (66.5)	1035^e^ (67.8)	88.5 (87–90)	0.79 (0.76–0.82)	Substantial	98 (9.7)	78 (15.2)	91 (89–92)	84 (81–87)	92 (90–94)	81 (77–84)
Conclusions (normal)	485 (31.7)	467 (30.6)	83.6 (82–86)	0.71 (0.68–0.75)	Substantial	134 (27.7)	116 (11.1)	75 (71–79)	87 (85–89)	72 (68–76)	89 (87–91)
Conclusion (pathological)	1043 (68.3)	1061 (69.4)				116 (11.1)	134 (27.7)	87 (85–89)	75 (71–79)	89 (87–91)	72 (68–76)

%FP are expressed as FP/exams coded “yes” for the specified condition by EEG technologist. %FN are expressed as FN/exam coded “no” for the specified condition by EEG technologists.

*ACI* Agreement coefficient, *FN* false negative, *FP* false positive, *IEDs* interictal epileptiform discharges, *IRA* interrater agreement, *PA* percentage of agreement, *Se* sensibility, *Sp* specificity, *NPV* negative predictive value, *PDR* posterior dominant rhythm, *PPV* positive predictive value, *SE* status epilepticus.

^a^2 missing data.

^b^11 missing data.

^c^26 missing data.

^d^18 missing data.

^e^1 missing data.

We then investigated which factors might influence EEG technologists’ reading abilities (Table 3). In intubated patients, EEG technologists tended to over-diagnose SE (FP rate 8% vs 1.3%, p < 0.001, significant after BH correction), while seizure detection was not affected. In contrast, in patients with known epilepsy, EEG technologists detected SE accurately but presented significantly increased FP for seizure detection (2.9% versus 0.7%, p = 0.001, significant after BH correction). SE diagnosis showed a significantly reduced IRA in older patients (p < 0.001, significant after BH correction), with EEG technologists demonstrating more FP among patients older than 60 years (3.3% versus 0.7%, p < 0.001, significant after BH correction). Patient cooperation significantly influenced EEG technologists’ performances. In poorly cooperating patients, EEG technologists displayed higher rates of FN seizure detection and higher FN and FP SE detection compared with cooperative patients (for all, p < 0.001 significant after BH correction). Among patients with a known history of craniotomy and among patients older than 60 years, the global conclusion showed significantly fewer FN results (i.e., EEG incorrectly coded as “pathological” by EEG technologists; for both p < 0.001, significant after BH correction). Similarly, poorer cooperation was associated with lower global conclusion rates. EEG indication (primary epileptic versus non-epileptic) and patient´s gender did not appear to affect the various studied parameters.

**Table 3:** EEG technologists’ reading performances stratified by potential cofounders.

	Craniotomy			Intubation			Known Epilepsy			Age > 60 years			Collaboration			
	Yes	No	p	Yes	No	p	Yes	No	p	Yes	No	p	Good	Moderate	Poor	p
**Seizures (yes)**																
Agreement, N (%)	317 (96.7)	1166 (97.3)	0.6	108 (95.6)	1376 (97.2)	0.3	552 (95.5)	923 (98.1)	**0.004**	644 (97.0)	839 (97.2)	0.9	1016 (97.8)^a^	197^a,b^ (97.5)^a,b^	271 (94.4)^b^	**0.01**
FP, N (%)	4 (1.2)	20 (1.7)	0.8	1 (0.9)	23 (1.6)	1	17 (2.9)	7 (0.7)	**0.001**	10 (1.5)	14 (1.6)	1	16 (1.5)	2 (1.0)	6 (2.0)	0.8
FN, N (%)	7 (2.3)	13 (1.7)	0.2	4 (3.5)	16 (1.1)	0.05	9 (1.6)	11 (1.2)	0.5	10 (1.5)	10 (1.2)	0.7	7 (0.7)^a^	3 (1.5)^a,b^	10 (3.5)^b^	**< 0.001**
**SE (yes)**																
Agreement, N (%)	314 (95.7)	1167 (97.3)	0.1	100 (88.5)	1382 (97.7)	**< 0.001**	561 (97.1)	921 (97.0)	1	632 (95.2)	849 (98.4)	**< 0.001**	1029 (99.0)^a^	196 (97.0)^b^	257 (89.6)^c^	**< 0.001**
FP, N (%)	9 (2.7)	19 (1.6)	0.2	9 (8.0)	19 (1.3)	**< 0.001**	11 (2.0)	17 (1.8)	0.8	22 (3.3)	6 (0.7)	**< 0.001**	7 (0.7)^a^	4 (2.0)^a,b^	17 (5.9)^b^	**< 0.001**
FN, N (%)	5 (1.5)	13 (1.1)	0.6	4 (3.5)	14 (1.0)	0.04	6 (1.0)	12 (1.3)	0.8	10 (1.5)	8 (0.9	0.3	3 (0.3)^a^	2 (1.0)^a^	13 (4.5)^b^	**< 0.001**
**Slowing (yes)**																
Agreement, N (%)	270 (82.3)	907 (75.7)	**0.01**	95 (80.5)	1087 (76.8)	0.4	434 (75.1)	744 (78.3)	0.1	526 (79.2)	651 (75.4)	0.0	763 (73.4)^a^	178 (88.1)^b^	237 (82.6)^c^	**< 0.001**
FP, N (%)	22 (6.8)	140 (11.7)	**0.008**	17 (15.6)	147 (10.4)	0.1	58 (10.1)	106 (11.2)	0.6	69 (10.4)	95 (11.1)	0.7	116 (11.2)	13 (6.4)	35 (12.4)	0.07
FN, N (%)	33 (10.1)	142 (11.9)	0.4	1 (0.9)	172 (12.2)	**< 0.001**	77 (13.4)	96 (10.1)	0.06	66 (9.9)	107 (12.5)	0.1	152 (14.7)^a^	11 (5.5)^b^	10 (3.5)^b^	**< 0.001**
**Alpha PDR (yes)**																
Agreement, N (%)	282 (86.0)	1067 (89.1)	0.1	95 (84.1)	1255 (88.8)	0.1	510 (88.2)	840 (88.6)	0.9	572 (86.3)	777 (90.1)	0.02	948 (91.4)^a^	159 (78.7)^b^	243 (84.7)^b^	**< 0.001**
FP, N (%)	23 (7.0)	55 (4.6)	0.09	13 (11.5)	65 (4.6)	**0.006**	30 (5.2)	48 (5.1)	0.9	32 (4.8)	46 (5.3)	0.7	37 (3.6)^a^	15 (7.4b)^b^	26 (9.1)^b^	**< 0.001**
FN, N (%)	23 (7.0)	75 (6.3)	0.6	5 (4.4)	93 (6.6)	0.5	38 (6.6)	60 (6.3)	0.9	59 (8.9)	39 (4.5)	**< 0.001**	52 (5.0)^a^	28 (13.9)^b^	18 (6.3)^a^	**< 0.001**
**Conclusion (normal)**																
Agreement, N (%)	294 (89.6)	983 (82.0)	**< 0.001**	113 (100)	1,165 (82.3)	–	477 (82.5)	801 (84.3)	0.39	581 (87.5)	696 (80.7)	**< 0.001**	810 (78.0)^a^	187 (92.6)^b^	281 (97.9)^c^	**< 0.001**
FP, N (%)	24 (7.3)	110 (9.2)	0.3	0	134 (9.5)	–	51 (8.8)	83 (8.7)	1	56 (8.4)	78 (9.04)	0.7	120 (11.6)^a^	10 (5.0)^b^	4 (1.4)^c^	**< 0.001**
FN, N (%)	10 (3.1)	106 (8.8)	**< 0.001**	0	116 (8.2)	–	50 (8.7)	66 (6.9)	0.2	27 (4.1)	89 (10.3)	**< 0.001**	109 (10.5)^a^	5 (2.5)^b^	1 (0.7)^b^	**< 0.001**

%FP are expressed as FP/exams coded “yes” for the specified condition by EEG technologist. %FN are expressed as FN/exam coded “no” for the specified condition by EEG technologists.

In bold: significant after Benjamini-Hochberg (BH) correction (15 comparisons).

*FN* False negative, *FP* false positive, *PDR* posterior dominant rhythm.

^a,b,c^Post-hoc analysis, performed using pairwise Fisher’s exact with BH correction. Groups sharing a letter are not statistically different.

Among the 725 patients with epilepsy diagnoses, epilepsy etiology did not influence seizure detection or the assessment of PDR or slowing. However, epilepsy etiology significantly altered the SE detection agreement rate (p = 0.002, significant after correction for multiple comparison), with neurophysiologist and EEG technologists presenting 97.8% agreement (n = 81) for patients with genetic epilepsy, 99.5% (n = 194) for patients with epilepsy of unknown etiology, and 94.6% (n = 421) among patients with symptomatic epilepsy. Global conclusion presented with significantly lower FN rates (i.e., EEG incorrectly coded as “pathological” by EEG technologists) among patients with symptomatic etiologies (p < 0.001; FN: 12.2%, n = 10 in genetic epilepsy; 14.9%, n = 29 in unknown etiologies; and 4.0%, n = 18 in symptomatic epilepsy).

Among patients who had previously experienced at least one epileptic seizure, none of the investigated EEG features significantly differed according to seizure semiology. Among patients with focal seizures, the EEG technologists’ reading performances, particularly for the detection of seizures, SE, or IEDs, did not differ according to the epileptic focus.

To check for a training effect, we divided our data into two time-periods (EEGs recorded during the 1st and the 2nd half of the study). We did not observe any effect regarding the overall conclusions, presence of seizure, slowing, or preserved PDR.

## Discussion

To our knowledge, this is the first study to prospectively investigate the reliability of EEG technologists’ reading performances in a large and heterogeneous population. EEG-technologists and neurophysiologists presented “substantial” IRA for overall EEG conclusion and “almost-perfect” IRA for seizures and SE detection. EEG technologists were able to identify normal EEG patterns with good sensitivity and specificity and detected ictal features (SE and seizures) with excellent NPV but low PPV. Intubation, older age, known epilepsy history, and poor cooperation might potentially alter the EEG technologists’ reading performances.

The IRA for SE and seizure detection was “almost perfect” (PA: 97% and AC1: 0.97 for both). A Canadian study investigated the IRA between experts and 16 neurological residents with brief training in ACNS terminology^13^. They reported “almost perfect” IRA for seizures but with slightly lower PA and Gwet-AC1 values than those found in our study (PA: 86.4%, AC1: 0.82). Although comparisons between studies should always be made with caution, these results suggested that, at least for ictal pattern detection, EEG technologists’ performances were similar to those of neurological residents. In many centers, in the absence of immediate availability of a certified neurophysiologist (for example at night or during weekends) clinical decisions are made upon neurological residents’ conclusion. Our results suggest that EEG-technologists conclusion might be similarly used.The identification of IEDs was associated with 78% PA and “substantial” IRA, which were in line with the results of a recent study investigating IED detection reliability among experts; who reported 80.9% PA and 69.4% kappa for the determination of whether an EEG contained any IEDs^11^. One study investigating the performance of automatic spike detection used three senior EEG technologists as alternate gold standards^21^. Although the IRA among readers was not formally assessed in that study, only 13.2% of the IEDs were detected by all three readers with FP/min ranging between 0.80 ± 1.61 and 1.99 ± 5.15, and sensitivity ranging between 40% and 51.5%^21^. Because the design of that study was very different from ours, the comparison between these results and our IED detection sensitivity proves difficult.

One striking result was the high specificity demonstrated by EEG technologists for the identification of ictal patterns. EEG technologists were able to diagnose seizures and SE with extremely low FN rates, suggesting that the physicians in charge can be reassured when EEG technologists report the absence of such patterns. However, this high NPV was counterbalanced by a low PPV, which should warn treating neurologists against starting any medications prior to examining the EEG for themselves. Among our cohort, the FP rate for seizures and SE diagnoses was above 50%. EEG overdiagnosis can lead to inappropriate epilepsy diagnoses, which is a well-known problem, even among neurologists, with up to 30% of the patients who are referred to epilepsy centers for refractory seizures eventually being diagnosed with no evidence of epilepsy^22–27^. Few data are available regarding the EEG reading accuracy of non-neurophysiologists. ICU physicians demonstrated limited (approximately 50%) sensitivity but good specificity (approximately 88%) for the identification of seizures^28,29^. However, because these studies focused on specific ICU populations (prognostication after cardiac arrest and recent clinical seizures), used simplified cEEG montages, and detected seizures based on amplitude-integrated EEGs (aEEGs), their results are difficult to compare with ours. The diagnosis of non-convulsive SE (NCSE) has been reported to be challenging, with experienced neurophysiologists presenting only “moderate” IRA and 47% PPV^30^. Unfortunately, because SE type was not recorded for study purposes in our population, we were unable to assess the contribution of NCSE to our FP rate.

We identified several factors that might influence the performances of EEG technologists and possibly other EEG readers. Patients with a known history of epilepsy presented with higher FP rates for seizure diagnoses, which is not surprising as “looking too hard” is a well-known cause of EEG overreading. The identification and analysis of overread patterns were beyond the scope of this study. Previous studies described “wicket spikes” and fluctuations in background activity with temporal phase reversal as the most frequently overread patterns^23,25,31^. Agreement regarding SE diagnoses was significantly lower among older patients. Several hypotheses can be made to explain this finding. First, NCSE, which is known to be more challenging to diagnose, is more common in the elderly^32^. Second, both ictal manifestations and interictal EEG findings are known to change with age, making the diagnosis of ictal features potentially more difficult among the older population^33^. However, the identification of seizures and IEDs was not altered by age in the present study. Additionally, the differential diagnosis of seizures or SE in older patients is particularly wide, and IEDs have been reported in up to 30% of patients with nonepileptic events, often leading to the inappropriate prescription of antiseizure medication^26,32,33^.

EEG indication goes beyond epilepsy, seizure diagnosis, or SE assessment. Around half of the EEGs were asked for non-epileptic reasons such as prognostication in patients with disorder of consciousness. As patients outcome were not available in the present study, the prognostication ability between EEG-technologist and neurophysiologist could unfortunately not be assessed. However, EEG-technologists reading ability, including features involved in prognostication assessment such as the presence of slowing or preservation of PDR, did not differ between EEG recorded for epileptic or non-epileptic reasons, suggesting that EEG-technologists may also contribute to these patients’ management.

In contrast with most previous studies investigating IRA, EEG readings were performed under “real-life conditions” in this study. Therefore, EEG technicians did not receive any specific training for the purposes of the study. Furthermore, as usual, EEG technicians read the EEGs at the patient’s bedside during the recording. Therefore, in addition to the EEG interpretation, they were also responsible for the patient’s care and the technical aspects of the recording. Furthermore, they had a limited amount of time (duration of the recording) to finalize their interpretations. On the other hand, neurophysiologists could read EEGs at their own rhythm.

These likely influenced our results and should be taken into account when interpreting them. In cases of doubt, without time to extensively examine the recording, EEG-technologists may have erred on the side of “overinterpretation” to draw neurophysiologists’ attention, contributing to the high FP rate. However, this “real-life design” might improve the clinical relevance in the everyday practice of our results.

In the absence of baseline evaluation, strong conclusions regarding a potential benefit of a checklist in routine clinical practice cannot be drawn. We did not observe any effect regarding the overall conclusions, presence of seizure, slowing, or preserved PDR. Furthermore, standardized EEG assessment, in which the readers were asked to assess specific EEG-features by choosing from a list of pre-defined terms, had already demonstrated to improve IRA^10,34–36^. Similarly, in other medical fields, it has been demonstrated that standardized assessment with use of predefined terms contributes to higher IRA^37^.

Some limitations of this study must be acknowledged. Our study included only routine EEG, with low numbers of SE and seizure recordings. This low number of ictal-events, with therefore low numbers of FN and FP, must be considered during the interpretation of our results, especially the analysis regarding potentials factors that may impair EEG-technologists reading performances. Furthermore, during cEEG, a “learning effect” may exist that would consequently lower the false positive rate. Because all EEG readers in this study (both neurophysiologists and EEG technologists) worked at a single academic center, our results do not necessarily represent the diversity of skills and knowledge among the community. Furthermore, all EEG-technologists at our center were certified in neurophysiological diagnostics (three years of specialized training including both theoretical and practical formation). As EEG technologist training varies between countries, this may impair the generalization of our results. Furthermore, our data came from one single tertiary hospital with specialized neuro-ICU and referral hospital for epilepsy surgery and neuro-oncological management. The proportion of patients who underwent prior craniotomy is therefore higher than the one expected in other centers without a neurosurgery department on site*.*One obvious limitation is the absence of a “true gold-standard test.” Interpretations by our certified neurophysiologists were considered to be correct without further formal assessments and without any “second-look” of EEG with discrepant interpretation between neurophysiologists and technologists. However, as part of the standard care at our institution, recordings with doubtful findings are discussed among various interpreters until a consensus is reached. Finally, as previously mentioned, the design of the study did not allow for the determination of which readers disagreed with specific EEGs.

## Conclusion

EEG indications are continually expanding, and the use of cEEG has increased. In this context, trained EEG technologists who are able to identify and recognize EEG abnormalities are essential, allowing them to alert physicians for timely interpretations and take necessary clinical actions. Indeed, credentialed EEG technologists have demonstrated the ability to improve patient management and outcomes^38^. In our study, EEG technologists demonstrated a “moderate” to “almost-perfect” IRA with neurophysiologists. EEG technologists were able to identify pathological features, especially ictal EEGs (SE, seizures), with almost no FNs, at the cost of a relatively high FP rate, the latter might be due to an “overinterpretation” by EEG technologists to draw neurophysiologists' attention. Physicians in charge should be able to rely on EEG technologists’ initial interpretations when they report the absence of ictal patterns but should always verify the EEG traces before taking medical action when ictal patterns are reported.

## Supplementary Information

Supplementary Information.

Supplementary Figure 1. EEG interpretation sheet. The left column was first filled by EEG-technologists during EEG recording and the right column was completed by neurophysiologists blinded to the EEG-technologists interpretation (two sheets pro patients were used). IEDs = Interictal Epileptiform discharges.

## Data Availability

Anonymized data can be shared upon reasonable request by the corresponding author.

## Code availability

All code used is available as part of SPSS, version 27 (IBM Corp., Armonk, NY, USA) and/or R, version 4.0.0 (R Foundation for Statistical Computing, Vienna, Austria).
