# Supplementary material for: Prospective evaluation of interrater agreement between EEG technologists and neurophysiologists
Source: Sci Rep. 2021 Jun 28;11:13406. doi: 10.1038/s41598-021-92827-3 (PMC8238944; doi:10.1038/s41598-021-92827-3)
Supplement: Supplementary file 1 — Supplementary Information. [file 41598_2021_92827_MOESM1_ESM.pdf]

Patient's Name<br><br>PID	Date: ______________________<br>Age______(Years)<br><br>Gender: W ☐(0) M ☐(1)	Diagnoses: _______________________<br>Epilepsy yes ☐(1) no ☐(0)<br>Sleep deprivation yes ☐(1) no ☐(0)

VIGILANCE	awake ☐(3)<br>somnolent (noise)<br>☐(2) stuporous<br>(pain) ☐(1)<br>comatose ☐(0)	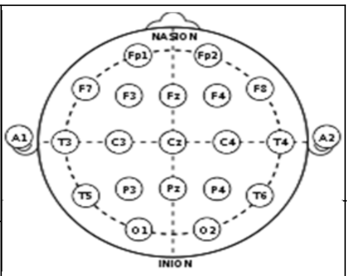
COLLABORATION	good ☐(2) average ☐(1) poor ☐(0)<br><br>Comments:	Craniotomy Yes ☐(1) No ☐(0)<br>Please fill craniotomy site
SLEEP	Yes ☐(1) No ☐(0)<br><br>**if yes** → Sleep stage: I ☐(1) II ☐(1) III ☐(1) REM ☐(1)	Trepanation ☐(1) ☐(0)<br>Sleep stage ☐(1) ☐(0)<br>I☐(1) II☐(1) III☐(1) REM☐(1)
HYPERVENTILATION	Yes ☐(1) No ☐(0)<br>**if yes** →<br>cooperation: good ☐(2) average ☐(1) poor ☐(0)<br>generalized slowing ☐(1)<br>focal slowing ☐(1)<br>IEDs ☐(1)	HV<br>generalized slowing<br>☐(1)<br>focal slowing ☐(1)<br>IEDs ☐(1)
PHOTIC STIMULATION	Yes ☐(1) No ☐(0)<br>**if yes** → Photic driving ☐(1)<br>Photoparoxysmal reaction ☐(1)	Photic driving ☐ (1)<br><br>Photoparoxysmal reaction ☐(1)
BACKGROUND ACTIVITY	Frequency: ___________Hz<br>Bi-occipital : No ☐(0)<br>Symmetric: No ☐(0)<br>Reactive: No ☐(0)	______Hz<br><br>bi-occ. ☐(0)<br>Symm. ☐(0)<br>Reactive ☐(0)
PATHOLOGICAL FINDINGS	<u>**Slowing**</u>: Yes ☐(1) No ☐(0)<br>**If yes** →<br>Focal : yes ☐(1)<br>Intermittent ☐(1) Continuous ☐(1)<br>Hemispheric: yes ☐(1)<br>Intermittent ☐(1) Continuous ☐(1)<br>Generalized: yes ☐(1)<br>Intermittent ☐(1) Continuous ☐(1)	Slowing ☐ (1) ☐(0)<br><br>Focal ☐ (1)<br>-intermittent ☐(1)<br>- continuous ☐(1)<br><br>Focal ☐(1)<br>- intermittent ☐(1)<br>- continuous ☐(1)<br><br>Generalized ☐ (1)<br>- intermittent ☐(1)<br>- continuous ☐(1)
	<u>**IEDs**</u>: Yes ☐(1) No ☐(0)<br>**If yes** →<br>focal yes☐(1)<br>multifocal yes ☐(1)<br>generalized yes ☐(1)	IEDs ☐ (1) ☐(0)<br><br>focal ☐(1)<br>multifocal ☐(1)<br>generalized ☐(1)
	**Periodic pattern** : yes ☐(1)<br>**Seizure**: yes ☐(1)<br>**Status epilepticus** : yes ☐(1)	Periodic patterns. ☐(1)<br><br>Seizure ☐(1)<br><br>Status epilepticus ☐(1)
Global Conclusions	EEG- technologist:<br>Normal : ☐(0)<br>Pathological: ☐(1)	Physician:<br><br>Normal ☐(0)<br>Pathological ☐(1)
