# Supplementary material for: Prospective evaluation of interrater agreement between EEG technologists and neurophysiologists
Source: Sci Rep. 2021 Jun 28;11:13406. doi: 10.1038/s41598-021-92827-3 (PMC8238944; doi:10.1038/s41598-021-92827-3)

**Supplemental Materials**

**Supplemental figure 1 : EEG interpretation sheet.**

The left column was first filled by EEG-technologists during EEG recording and the right column was completed by neurophysiologists blinded to the EEG-technologists interpretation (two sheets pro patients were used).

IEDs = Interictal Epileptiform discharges.


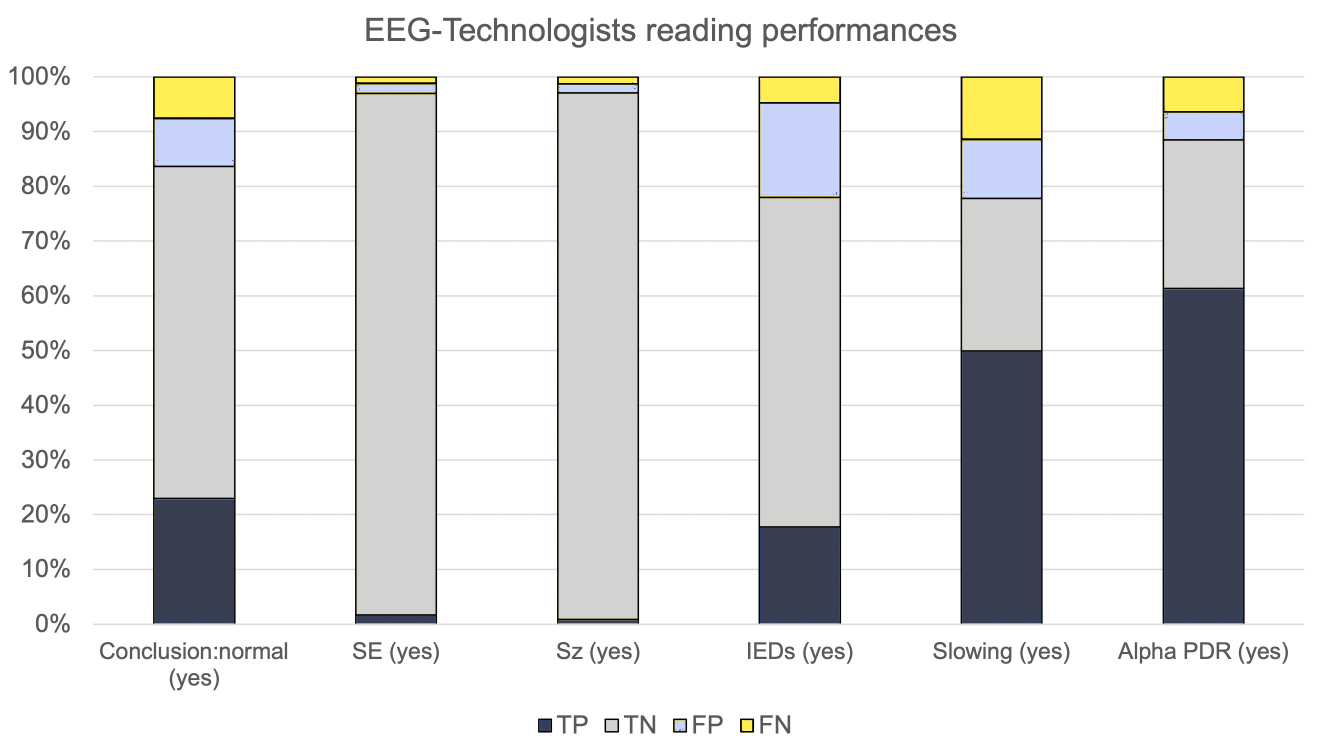

Supplement: Supplementary file 2 — Supplementary Figure 1. EEG interpretation sheet. The left column was first filled by EEG-technologists during EEG recording and the right column was completed by neurophysiologists blinded to the EEG-technologists interpretation (two sheets pro patients were used). IEDs = Interictal Epileptiform discharges. [file 41598_2021_92827_MOESM2_ESM.docx]
